# Validation of the flourishing scale and scale of positive and negative experience in a Chinese community sample

**DOI:** 10.1371/journal.pone.0181616

**Published:** 2017-08-03

**Authors:** Kwok Kit Tong, Yuan Yuan Wang

**Affiliations:** Department of Psychology, University of Macau, Macau, China; IRCCS Istituto Auxologico Italiano, ITALY

## Abstract

Diener and colleagues (2010) created concise assessment tools to study flourishing and Positive and Negative emotions. The two scales had been validated with English-speaking and German-speaking participants. Nevertheless, its applicability for Chinese is not well established. The current study validated the two scales in traditional Chinese, using a randomly selected community sample. Both the flourishing scale and the Positive and Negative Emotions scale showed an adequate fit. We also found that gender and social status had an influence on flourishing.

## Introduction

Psychological well-being and subjective well-being studies have attracted increasing number research in recent decades [1–3]. Diener (1984) defined subjective well-being consists of both emotional and cognitive appraisals of the quality of one’s own life [4]. Researchers do not have a consensus on its operational definition and hence the measurement of subjective well-being has always been a dynamic research area [4–8]. On the other hand, drawing from studies on self-actualization, Ryff (1989) made a distinction between eudaimonia well-being and hedonic well-being in her seminal paper on psychological well-being [3] and it generated lots of discussions and arguments in the field [9–13]. There is no universal agreement on the distinction between eudaimonia well-being and hedonic well-being. To some extent, eudaimonia well-being focuses on pleasure attainment and pain avoidance, while eudaimonia well-being focuses on the degree of self-realization [11]. Despite all, many researchers agree that hedonic well-being and eudaimonia well-being are both important dimensions of well-being. In his book *Flourish*, Seligman (2011) identified measureable elements such as Meaning, and Achievement to tap flourishing [14]. Some researchers also suggested an affective component [4–5, 15]. In Diener (1984)’s early formulation, the subjective well-being is consisted of life satisfaction, positive affect and negative affect [4]. Researchers also developed measures such as Positive and Negative Affect Schedule [15], Affect Balance Scale [5], and Hedonic Balance Scale [16] to tap a range of emotions and feelings. It should be noted that there are other definitions and researchers are yet to have an agreement on definitions and measurement [17].

From a psychosocial perceptive, Diener and colleagues (2010) conceptualized flourishing as the fulfilment of the needs of competence, relatedness and self-acceptance as well as the possession of psychological capital such as flow and engagement [18]. In concert with the positive psychology movement, prominence researchers in the field have shown growing interests in this research domain [9, 14, 18]. Drawing from past research on social-psychological prosperity and psychological capital, Diener et al. (2010) created an 8-item scale, the Flourishing Scale (FS) and Scale of Positive and Negative Experience (SPANE) to assess broader concept of well-being [18]. The FS covers aspects of social-psychological prosperity such as social relationships, having a purposeful and meaningful life, being engaged and interested in one’s activities, tapping self-respect and optimism, feeling competent and capable, while SPANE covers aspects of positive and negative feelings. In comparison with some existing two-dimensional measurements for well-being, Diener et al. (2010) contended that SPANE is able to tape a wide range of emotions and feelings with smaller number of items [18].

In developing FS and SPANE, Diener and colleagues (2010) recruited 689 participants from five universities in the States and the Singapore and they showed that both scales have good psychometric properties and they are also congruent with validated well-being measurement such as the Satisfaction with Life scale [18]. The FS scales have been validated in Portuguese [19], English (in New Zealand [20] and Canada[21]) and German (in German) [22] and the SPANE has also been validated in a Chinese work sample recently [23]. Although the FS and SPANE show good psychometric properties in validation studies, ongoing works are needed to confirm their validity as well as factor structure since most studies in FS and SPANE utilizes convenience sample such as students and employees, undermining the applicability of the measurement to the community. Research in Chinese well-being is relatively sparse compared to the West because of the lack of validated measurement of well-being measurement [24]. The FS has not been tested rigorously on Chinese societies, with different levels of economic development, except for studies reported by Tang Duan, Wang & Liu, (2016) and Duan and Xie (2016) [25–26]. Tang et al. (2016) tested and verified a simplified Chinese version of the FS in communities in the south west region in China and Duan and Xie (2016) tested the FS on Chinese adolescents recruited in middle and high schools [25–26]. Their results indicated that the FS has good psychological properties and the application of it in Chinese societies is promising.

The present study has three objectives. The first objective is to validate the FS scale in economically developed Chinese societies, such as Taiwan, Hong Kong and Macau, in traditional Chinese (c.f., simplified Chinese characters are used in mainland China and the characters contain fewer strokes and are easier to write). We also test their relations to existing well-being measurements such as Diener’s satisfaction with life scale. The second objective is to evaluate FS and SPANE using a community sample using a two-stage sampling procedure in a phone poll so that the applicability of the measurements can be generalized beyond school and organizational settings. The third objective is to explore FS’s relation to personal characteristics such as perceived social status and marriage status. The information is particularly meaningful using a community sample.

## Method

### Participants

A total of 1008 people were interviewed by phone in Macao. They were all informed of their rights of withdrawal. There were 548 females and 460 males, with age ranged from 18 to 85 years old (Mean = 40.26, SD = 16.79). The median monthly income level was MOP16000 (USD2000).). The sample characteristics were listed in Table 1.

**Table 1:** Summary of the sample characteristics (N = 1008).

		Total (%)			
		N = 1008	Valid%	Male (%)	Female (%)
Sex					
	Male	460	45.6		
	Female	548	54.4		
Age	24 or below	237	23.5	23.7	23.4
	25–34	195	19.3	21.3	17.7
	35–44	128	12.7	11.1	14.1
	45–54	185	18.4	17.2	19.3
	55–64	134	13.3	14.3	12.4
	65 and above	129	12.8	12.4	13.1
Education					
	Uneducated	28	2.8	3.1	2.6
	Primary school	110	11.0	10.9	11.0
	Junior high school	173	17.3	17.3	17.2
	Senior high school	282	28.1	28.7	27.7
	College and above	409	40.8	40.0	41.5
Job status					
	Full-time	572	57.0	61.7	53.1
	Part-time only	84	8.4	9.4	7.5
	No (housework duties)	67	6.7	0.4	11.9
	No (retired)	108	10.8	11.6	10.1
	No (student)	124	12.4	11.8	12.8
	unemployed	48	4.8	5.0	4.6
Marital status					
	Single	392	39.2	39.8	38.7
	Married	595	59.5	59.3	59.7
	Divorced	3	0.3	0.2	0.4
	Partner deceased	10	1.0	0.7	1.3
Perceived Social Class					
	Lower class	108	11.0	12.1	10.0
	Lower-middle class	293	29.7	34.3	25.8
	Middle class	488	49.5	45.1	53.3
	Upper-middle class	90	9.1	8.1	10.0
	Upper class	7	0.7	0.4	0.9

### Materials

In addition to social-demographic information, the following instruments were included in the survey and they were translated into traditional Chinese by a bilingual translator and then back-translated to English by another translator to ensure the correctness of the translation.

#### The flourishing scale

The FS is a 7-point Likert scale with 8 items (from strong disagreement to strong agreement) that measures participant’s self-perceived success in areas such as relationships, self-esteem, purpose, and optimism [18]. Its sum score ranges from the lowest 8 to the highest 56. One sample item of the FS is “My social relationships are supportive and rewarding.”

#### Scale of positive and negative experience

SPANE is a 5-point Likert scale (from never to always) with 12 items, which includes 6 items (e.g., positive, good, pleasant) assessing positive feelings (SPANE-P) and 6 items (e.g., negative, bad, unpleasant) assessing negative feelings (SPANE-N) [18]. The scores of SPANE-P and SPANE-N range from 6 to 30. The summed balanced between positive and negative feelings (SPANE-B) is the difference score between positive feeling items and negative feeling items and it ranges from -24 to 24.

#### Satisfaction with life scale (SWLS)

SWLS measures general life satisfaction [27]. It contains five 7-point-scale items (e.g., “In most ways my life is close to my ideal.”).

#### Perceived physical health

This is a single item measurement on participant’s self-perception of physical health. It is measured on a 5-point scale, from *very unhealthy* to *very healthy*.

#### Perceived social class

This is a single item measurement on participant’s self-perceived social class, which is measured on a 5-point scale, range from *lower class*, *lower-middle class*, *middle class*, *upper-middle class*, and *upper class*.

#### Martial status

This is a single item measurement (single, married, others).

### Procedure

We collected the data by phone. A sample of residential phone numbers were randomly drawn from the local telephone directory and for each selected household, eligible participant were randomly selected based on the last birthday rule. We called back up to 5 times at different dates and different times for non-contact cases. The study was conducted anonymously. A verbal consent was obtained and the interviewees knew that they could quit and skip questions anytime. A written approval was not obtained because we contacted interviewees by phone. The willingness of participation was record by a yes/no screen question and participants who were not taken part was thanked and the interview terminated. The phone interview procedure and consent procedure of this study were approved by University of Macau committee on ethics. The average interview time was around 14 minutes.

## Results

### Descriptive statistics of the scales

The means and standard deviations for FS, SPANE, SWLS and perceived physical health were reported in Table 2.

**Table 2:** Descriptive statistics of the scales.

	N	Item Minimum	Item Maximum	Item Mean	Scale Mean	SD	Cronbach’s Alpha	Scale Range
FS (8 items)	876	4.39	4.91	4.605	36.84	8.47	.88	8 to 56
SPANE P (6 items)	953	3.39	3.71	3.50	21.00	4.39	.82	6 to 30
SPANE N (6 items)	948	2.03	2.64	2.28	13.66	4.82	.81	6 to 30
SPANE B (12 items)	931	/	/	/	7.31	7.34	.85	-24 to 24
SWLS (5 items)	915	3.54	4.08	3.78	18.89	5.79	.79	5 to 35
Perceived Physical Health	1002	1	5	3.47	/	.92	/	1 to 5

*Note*: The reliability of SPANE-B is based on reliability of difference score.

### Scale validity of the FS and SPANE

We consider the model to be acceptable based on the following criteria: the comparative fit index (CFI) is greater than .90 [28], the root mean square error of approximation (RMSEA) is less than .08 [29], the Tucker-Lewis Index is greater than .90 [30]. We also report AIC (Akaike’s Information Criteria) and BCC (the Browne-Cudeck Criteria) to inform model selection and a smaller number is preferred [31].

**Fig 1:**
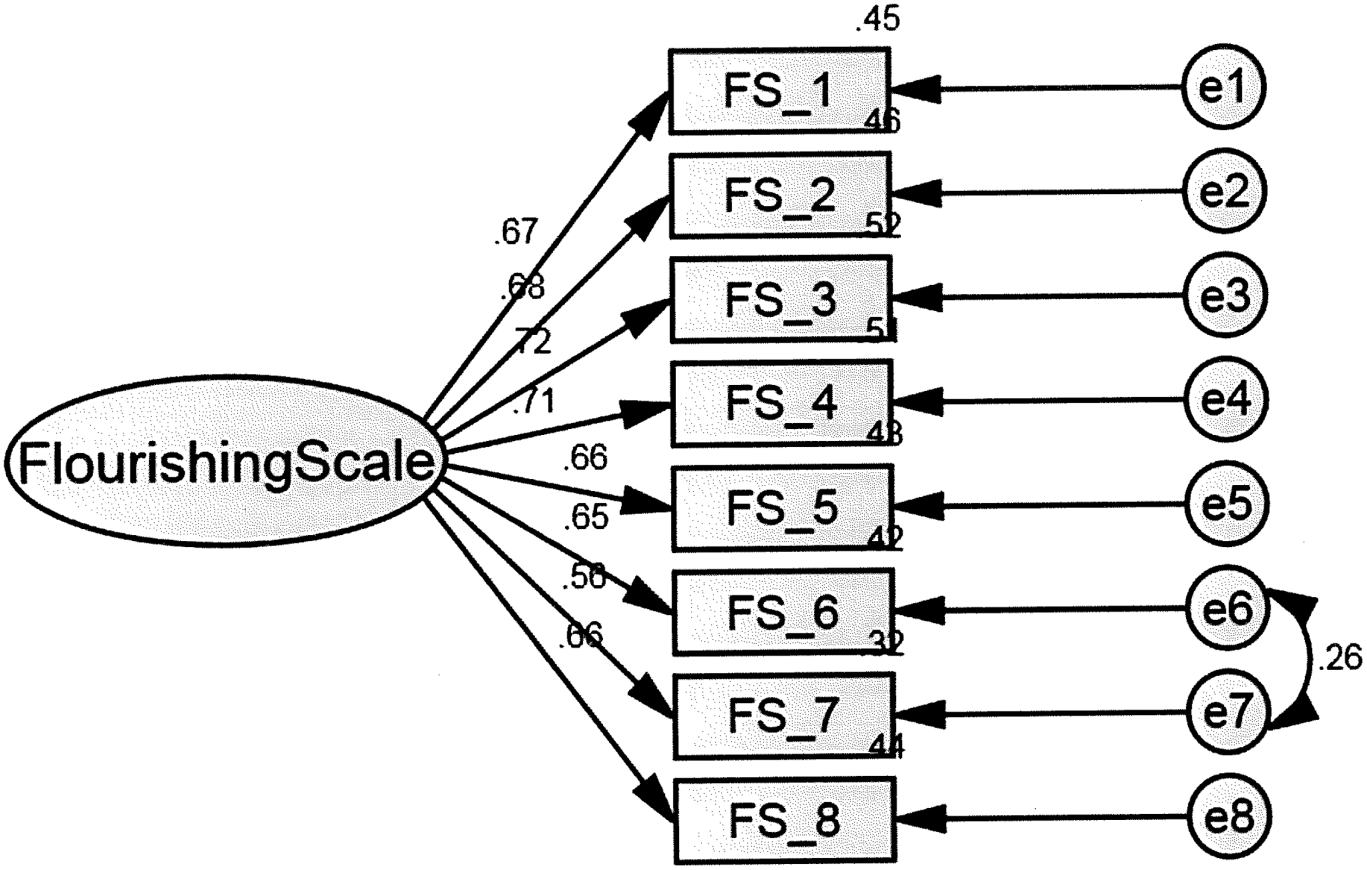
Flourishing scale. Confirmatory Factor Analysis of the Flourishing Scale.

Both Diener et al. (2010) and Silva and Caetano (2013) suggested that FS is a single dimension construct [18–19]. We conducted a confirmatory factor analysis on the traditional Chinese version of the FS by Amos 20, χ2 (N = 1008, df = 20) = 211.374, p< .01, RMSEA = .097, CFI = .933, TLI = .907, AIC = 243.37, BCC = 243.66. It indicated an inadequate fit. We examine the modification index and the single-factor model showed a better fit if we free one error covariance (item e6 “I am a good person and live a good live” and item e7 “I am optimistic about my future”), χ2 (N = 1008, df = 19) = 162.89, p < .01, RMSEA = .087, CFI = .949, TLI = .904, AIC = 212.89, BCC = 213.34 (Fig 1).

**Fig 2:**
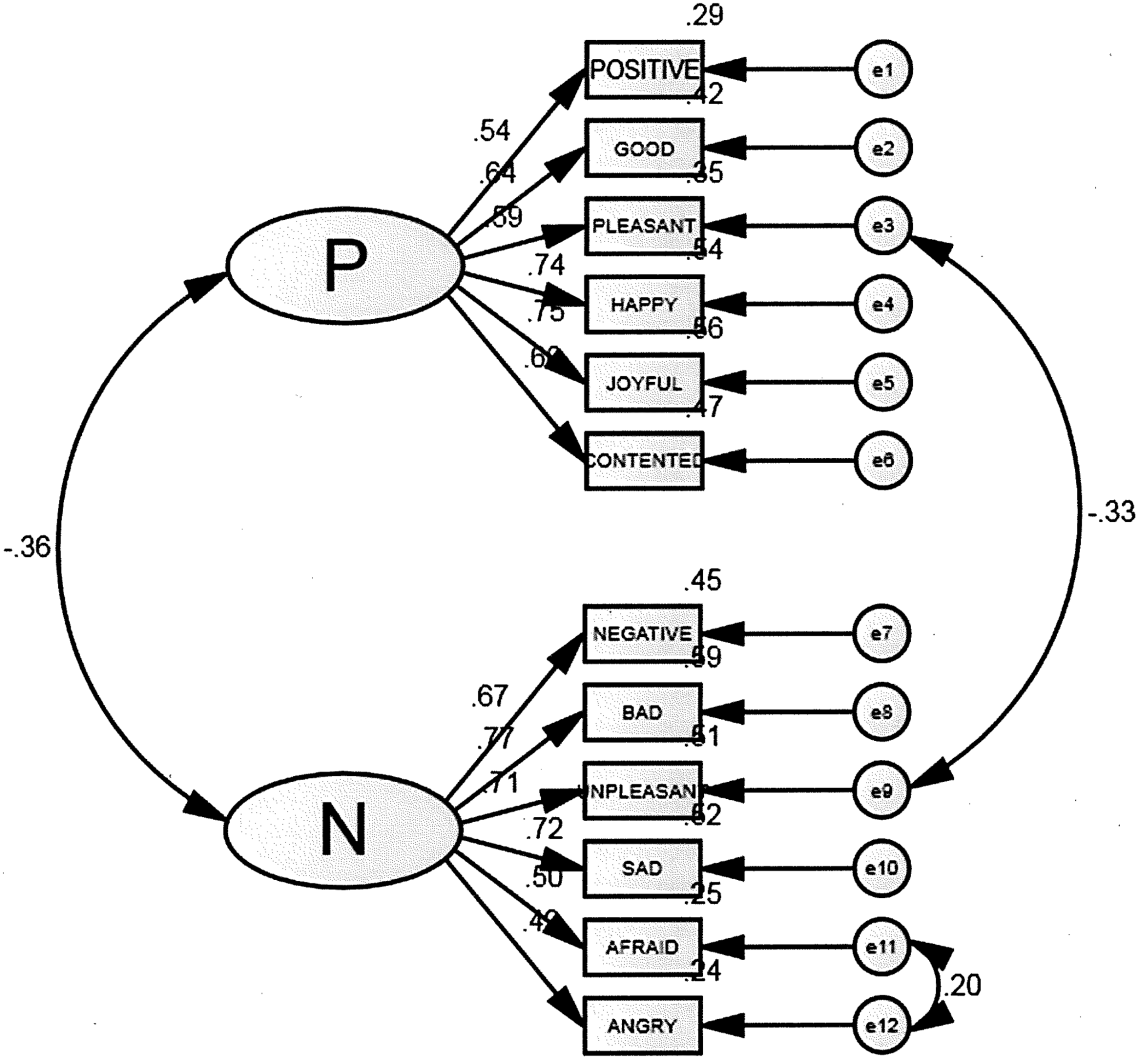
SPANE scale. Confirmatory Factor Analysis of the SPANE Scale.

We tested the two-dimensional SPANE model with CFA by using Amos 20, χ2 (N = 1008, df = 53) = 387.221, p< .001, RMSEA = .079, CFI = .912, TLI = .870, AIC = 461.221, BCC = 462.189. It indicated an inadequate fit. We examine the modification index and if we free two error covariances (item e3 Pleasant with items e9 Unpleasant, and item e11 Afraid with item e12 Angry), the model fit improved, χ2 (N = 1008, df = 51) = 275.203, p< .001, TLI = .910, CFI = .941, RMSEA = .066, AIC = 353.203, BCC = 354.223 (Fig 2). The modification is post-hoc and further studies are required to evaluate if we need to improve the items for Chinese respondents. Given that the indicators were related, we check if it may indicate a one-factor model. We tested the one-factor SPANE model with CFA using Amos 20, χ2 (N = 1008, df = 54) = 1639.627, p< .001, RMSEA = .171, CFI = .582 TLI = .397, AIC = 1711.627, BCC = 1712.569—indicating an inadequate fit. Given larger AIC and small CFI in the one factor model, SPANE was not a single dimensional construct.

### Properties of FS and SPANE

The Cronbach alphas for FS, SPANE-P, SPANE-N, SPANE-B and SWLS were .88, .82, .81, .85, and .79 respectively. The reliability of SPANE-B was based on reliability of the difference score [32]. The results showed that the internal consistency of the traditional Chinese versions of both FS and SPANE were acceptable.

To test the convergent validity, we examine the relation between FS, SWLS and perceived physical health (Table 3). FS was positively correlated with perceived physical health (r = .200, p< .01), SWLS (r = .494, p< .01), SPANE P (r = .426, p< .01), and SPANE B (r = .458, p< .01), and was negatively correlated with SPANE N (r = -.308, p< .01). SPANE (P, N, B) were significantly correlated with perceived physical health (r = .26, p< .01; r = -.19, p< .01; r = .28, p< .01 respectively), and SWLS (r = .398, p< .01; r = -.275, p< .01; r = .416, p< .01 respectively). The results were consistent with past studies.

**Table 3:** Inter-correlation table for FS, SPANE, body item and SWLS.

	FS	SPANE P	SPANE N	SPANE B	BODY ITEM	SWLS
FS	1	.426**	-.308**	.458**	.200**	.494**
SPANE P	.426**	1	-.272**	.776**	.260**	.398**
SPANE N	-.308**	-.272**	1	-.818**	-.190**	-.275**
SPANE B	.458**	.776**	-.818**	1	.280**	.416**
Perceived Physical Health	.200**	.260**	-.190**	.280**	1	.131**
SWLS	.494**	.398**	-.275**	.416**	.131**	1

Note:

**. Correlation is significant at the .01 level (2-tailed)

### Gender differences

Female participants scored significantly higher on FS (M = 37.66) than male participants (M = 35.89), with *t* (874) = 3.09, *p* < .01. Male participants scored significantly higher on perceived physical health (M = 3.58) than female participants (M = 3.38), with *t* (1000) = 3.36, *p* < .001. Males and females were not significantly different in SPANE (SPANE-P, SPANE-N and SPANE-B) and SWLS.

### Perceived social classes and marital status

ANOVA showed that perceived social classes affected the FS score, *F* (4, 857) = 12.14, *p* < .001. Post-hoc comparisons showed that participants from lower (M = 33.93) and lower middle social classes (M = 35.05) scored lower than middle (M = 37.50), upper middle class (M = 40.96) and upper class (M = 42.00) participants, while middle class participants also scored lower than upper middle class participants. On the other hand, The middle class (M = 21.52), the upper middle class (M = 23.49) and the upper class (M = 22.86) participants scored higher in SPANE-P, *F*(4, 933) = 19.532, *p* < .001 than the lower class (M = 18.79) and the lower middle class (M = 20.06) participants. Likewise, the lower class (M = 15.64) and the lower middle class (M = 14.30) participants scored higher in SPANE-N, *F*(4, 929) = 8.097, *p*< .001 than the middle class (M = 13.13) and the upper middle class (M = 12.57) participants The mean for the upper class participants (M = 12.86) was not significantly different from the others.

Marital status has an impact on SPANE-N, *F* (2, 939) = 8.255, *p* < .001. Post-hoc comparisons showed that married participants (M = 13.17) scored significantly lower than single participants (M = 14.38). Marital status had no effect on SPANE-P nor FS scores.

## Discussion

Similar to previous studies [18–20], the traditional Chinese version of FS and SPANE had acceptable reliability based on Henson (2001)’s criteria [33]. Analyzing the correlations among FS, SPANE and SWLS reveals that they have good convergent validity. Chan & Davey (2008) argued that tools in studying Chinese well-being were inadequate [24] and the current study fill in the research gap and it could facilitate future studies of well-being. In addition, our data suggested that FS is an single-dimensional construct while SPANE is two-dimensional. Our findings were in line with previous studies [2–3, 14]. Hence, the current study provided additional evidence regarding dimension and measurement of flourishing.

Interestingly, Diener et al., (2010) reported no evidence of gender difference on the FS [18], while Howell and Buro (2014) reported a gender difference [21]. In particular, they found that women scored higher than men on the FS. In line with their study, we also demonstrated that women scored higher on flourishing. Unfortunately, we failed to confirm Li et al. (2013)’s finding [23] on gender difference in SPANE. Although there is no widely accepted conclusion because of mixed findings in different studies, Arrosa and Gandelman (2016) recently argued that female tend to be happier than man worldwide [34]. Our findings support the view that women seem to be in an advantage. A local government report suggested that women’s social network was better in terms of the emotional supports given and the breadth of their social networks [35]. We suspect that enhanced social supports for female may contribute to a higher score in the FS in our study. On the other hand, participants with a higher perceived social class also achieved a higher FS and SPANE-P scores. Social resources are wealth, status, social ties and power linked to a person [36]. According to a government report [35], high SES Macao people had more resourceful social networks. We believe that the relation between social resources and the FS in Chinese societies may be a fruitful avenue to investigate the effect of gender and social status.

Although there were a few validation studies on the FS [19–22, 25–26], they did not utilize a phone poll to collect a probability sample representing the community. Hence, it was not certain whether the findings could be applied to people with different employment status or different age groups. The current study filled in the research gap and provided a support to the applicability of Diener et al. (2010)’s scale [18] in the community and enabled the generalizations of the findings to people with different backgrounds. The validation of the scales in a community sample could facilitate comparisons among social groups with different ages and social status.

We found that lower social status people scored lower in the FS, implying social status may have an impact on flourishing. In particular, people who had a lower social status are in a disadvantage. Further study is required to investigate the causes. In fact, we believe that the FS may be used to supplement existing measurements to evaluate if existing welfare policies can help those who are in need, usually with lower social status because the FS also measures domains that are not captured by socio-economic indicators and well-being measurements. On the other hand, to enhance equal opportunities for the disadvantaged, we may also take into account the flourishing dimensions suggested by Diener et al., (2010) [18] or other researchers. Another implication was that in previous studies with university samples, university students may be less likely to belong to the lower status group and the FS scores obtained from university samples may be an overestimation.

There were a few limitations in our study. The present study could not answer questions such as the issue of ethnicity on the FS and SPANE and whether the scale may or may not apply to minority groups. In additional, similar to other validations studies in the FS and SPANE, the present study did not take into account potential cultural differences and hence there was no unique dimension being explored nor proposed. In the present study, the FS and SPANE model are adequate if we have allowed a correlation between errors of two items respectively. It suggests that there may be a factor (or factors) that can account for the error variances of the two items (i.e., I am a good person and live a good life and I am optimistic about my future). For example, physical and social resources available may be factors that give rise to the relation. This is a post hoc explanation and future analysis is required. We need to pay attention to the applicability of the items and further studies would be required to explore if the items needed to be modified for a Chinese sample, and how. Theoretically, there is also a need to find out if there are culturally-specific dimensions on flourishing.

Rayo and Becker (2007) argued that the pursuit of happiness is the principal motivating factor in a person’s life [37]. With the largest population in the world, a fast growing economy, dynamics changes in its societies and the standard of living, the study of flourishing in Chinese societies are theoretically and practically interesting. A validated measure of well-being will largely promote and assist the development of positive psychology and happiness research. The current study, using a community sample, demonstrated the psychometric properties of the FS and SPANE, indicating they would be a vital tools in future well-being studies in Chinese societies.
